# M2-PRNet: multi-scale and multi-modal learning for protein–RNA binding affinity prediction

**DOI:** 10.1093/bioinformatics/btag614

**Published:** 2026-08-17

**Authors:** Junkai Wang, Gang Luo, Yunsong Yang, Zhilin Zhu, Min Li

**Affiliations:** School of Computer Science and Engineering, Central South University, Changsha 410083, China; School of Computer Science and Engineering, Central South University, Changsha 410083, China; School of Computer Science and Engineering, Central South University, Changsha 410083, China; School of Computer Science and Engineering, Central South University, Changsha 410083, China; School of Computer Science and Engineering, Central South University, Changsha 410083, China

## Abstract

**Motivation:**

Predicting protein–RNA binding affinity is crucial for understanding cellular regulation and advancing RNA-targeted drug discovery. However, this task remains challenging due to structural complexity, limited labeled data, and insufficient modeling of fine-grained interactions.

**Results:**

We propose M2-PRNet, a multi-scale and multi-modal framework that integrates atom-level graphs, residue-level graphs, and tri-view molecular representations to capture complementary structural information. A cross-scale contrastive learning objective is introduced to align representations across different structural resolutions of the same complex. Under a clustering-based five-fold cross-validation setting on benchmark datasets, M2-PRNet achieves state-of-the-art performance. To further assess generalization under reduced sequence homology, we construct homology-aware RNA-cold, protein-cold, and dual-cold evaluations under a stricter 40% sequence identity threshold, where M2-PRNet maintains competitive performance. To account for conformational flexibility, we evaluate the model on MD150-1ns and an extended MD75-10ns subset, demonstrating stable performance under MD-derived structural perturbations. In addition, representative case studies suggest that M2-PRNet can highlight relevant RNA-binding regions and support preliminary discrimination between strong and weak binders when plausible complex structures are available. These results demonstrate the effectiveness of integrating multi-scale and multi-modal representations with cross-scale alignment for protein–RNA affinity prediction.

**Availability and implementation:**

The source code and datasets for M2-PRNet are freely available at https://github.com/CSUBioGroup/M2-PRNet.

## 1 Introduction

Protein–RNA interactions play fundamental roles in diverse biological processes, including post-transcriptional regulation, RNA splicing, translation, and subcellular localization (Corley *et al.* 2020, Zhou *et al.* 2020). With the rapid development of RNA-based therapeutics, such as mRNA vaccines, antisense oligonucleotides, and small interfering RNAs, accurate prediction of protein–RNA binding affinity has become increasingly important for drug discovery and functional annotation. Several RNA-based therapies have reached the clinic, highlighting the therapeutic relevance of protein–RNA interactions (Benson *et al.* 2018, Ray *et al.* 2023).

However, modeling protein–RNA binding remains challenging. Unlike protein–protein interactions, protein–RNA binding does not follow a single structural paradigm. While some interactions are mediated by well-defined RNA-binding domains, many involve intrinsically disordered regions, which are highly flexible and can adopt diverse conformations upon binding (Zhao *et al.* 2021). These interactions may form partially ordered or “fuzzy” complexes, making them difficult to characterize with a single static structure (Tompa and Fuxreiter 2008, Varadi *et al.* 2015). As a result, protein–RNA interactions exhibit significant structural variability, posing challenges for affinity prediction and generalization.

Experimental measurements of protein–RNA binding affinity are costly and time-consuming, and often fail to capture the full range of conformational diversity (Darnell 2010, Weeks 2010, Zhang *et al.* 2022). This limitation is further exacerbated by the dynamic and heterogeneous nature of RNA molecules, especially in multi-chain complexes. Consequently, computational methods have become essential for large-scale affinity prediction and screening.

Existing computational methods for protein–RNA binding affinity prediction can be broadly categorized into sequence-based, structure-based, and hybrid approaches. Sequence-based methods leverage primary sequences and benefit from efficiency and data availability, but lack structural awareness. Structure-based approaches incorporate atomic or interfacial features to improve interpretability, yet are highly dependent on structural quality. Hybrid methods that integrate sequence and structural information have improved predictive performance, but this gain often comes at the cost of increased model complexity.

Representative sequence-based models include PNAB (Yang and Deng 2019), which employs an ensemble of traditional regressors on handcrafted features, and DeePNAP (Pandey *et al.* 2024), which utilizes convolutional neural networks to learn from raw sequences. Structure-based methods such as PredPRBA (Deng *et al.* 2019) and PRdeltaGPred (Aletayeb *et al.* 2026) exploit interfacial structural features, while PRA-Pred (Harini *et al.* 2024) integrates interaction features through multivariate regression. Although these methods improve predictive performance, they often generalize poorly across diverse RNA conformations. Hybrid approaches further combine sequence and structural information. Notably, Copra (Han *et al.* 2025) integrates cross-modal sequence and structural features of proteins and RNAs. It employs a bi-scope pre-training strategy, including a cross-modal contrastive protein–RNA interaction task and a masked interface distance modeling task. However, existing methods still lack explicit modeling of multi-scale interaction mechanisms, limiting their effectiveness for structurally complex or flexible protein–RNA systems.

To address these challenges, we propose M2-PRNet, a multi-scale and multi-modal framework for protein–RNA binding affinity prediction. The model captures interactions across complementary structural resolutions, including atom-level graphs for fine-grained physicochemical interactions, residue-level graphs for intermediate structural semantics, and tri-view molecular representations for global binding geometry. These multi-scale features are integrated through a hierarchical gated fusion strategy, enabling the model to adaptively aggregate information across scales and modalities. In addition, a lightweight cross-scale contrastive objective aligns atom- and residue-level representations, encouraging consistency across different structural resolutions of the same complex.

We evaluate M2-PRNet on benchmark datasets, homology-aware cold-start splits, MD-derived structural perturbation sets, and representative case studies. Experiments on PRA310 and PRA201 demonstrate state-of-the-art performance under clustering-based five-fold cross-validation. Under stricter 40% sequence identity control, M2-PRNet maintains competitive performance in RNA-cold, protein-cold, and dual-cold evaluations. The model also remains stable on MD150-1ns and the extended MD75-10ns subset. Case studies on experimentally resolved complexes and a VEGF165 predicted-structure aptamer case further support its interpretability and applicability to predicted-structure. These results suggest that M2-PRNet provides an effective structure-based framework for protein–RNA affinity prediction and may support preliminary RNA-targeted candidate screening.

## 2 Materials and methods

### 2.1 Protein and RNA language models in bioinformatics

Language models trained on biological sequences have become widely used for bioinformatics tasks. Representative examples include ESM-2 (Rives *et al.* 2021) for proteins and RiNALMo (Penić *et al.* 2025) for RNA, which learn context-dependent embeddings from large sequence datasets. These embeddings capture patterns related to structure and function and are commonly used as input features for structural analysis, functional annotation, and interaction modeling without requiring explicit structural information (Jeliazkov *et al.* 2023). However, sequence-based embeddings alone may not fully capture the explicit 3D structural context, dynamic conformational variations, and fine-grained intermolecular interactions required for accurate protein–RNA affinity prediction, motivating the integration of structural and spatial representations.

### 2.2 Graph neural networks for molecular representations

Graph neural networks (GNNs) naturally model molecular structures by operating on atomistic graphs, capturing relational and spatial interactions critical for biological tasks. In RNA and protein–RNA modeling, geometric vector perceptron networks (GVP) (Liang *et al.* 2025) and equivariant GNNs (Boadu *et al.* 2023, Roche *et al.* 2023) have been used for 3D structure prediction and scoring, and Graph Transformer architectures such as those based on invariant point attention (IPA) in AlphaFold2 (Jumper *et al.* 2021, Yuan *et al.* 2022) effectively encode geometric context in structural representations. These studies motivate our use of multi-scale graph representations for protein–RNA binding affinity prediction. Despite their strength in modeling local geometric interactions, graph-based methods often focus primarily on atomistic or residue-level neighborhoods and may be limited in capturing complementary global structural patterns.

### 2.3 ConvNeXt for molecular structure representation

ConvNeXt (Woo *et al.* 2023) is a modern CNN architecture that captures local spatial patterns and hierarchical visual features, achieving competitive performance with Transformer-based models in image recognition (Liu *et al.* 2021). Image-based molecular representations have recently been used in prediction tasks, such as ImageMol (Zeng *et al.* 2022) and MolNexTR (Chen *et al.* 2024), and are increasingly applied in drug discovery (Li *et al.* 2024). For protein–RNA complexes, tri-view molecular images allow ConvNeXt to extract global spatial and shape-level features that are difficult to capture from sequence or graph representations alone. However, image-based representations may lose explicit molecular connectivity and fine-grained physicochemical interactions.

Taken together, these limitations motivate the development of a multi-scale and multi-modal framework that jointly leverages sequence, geometric, and image-level representations for more robust protein–RNA affinity prediction.

### 2.4 Multi-scale graph and image representation

#### 2.4.1 Atom-level subgraph

At the atom level, we construct a graph to capture local protein–RNA interactions. Protein pocket atoms are selected from residues containing at least one atom within 8 Å of any RNA atom, while all RNA atoms are retained. Edges are defined using both geometric and biochemical priors. Geometric proximity edges connect atom pairs within 8 Å, capturing local spatial contacts. Biochemical prior edges include RDKit-derived covalent bonds and interaction fingerprint (Bouysset and Fiorucci 2021) edges describing RNA–protein physicochemical interactions. To represent aromatic-ring interactions, virtual nodes are placed at ring centroids and connected to the corresponding ring atoms. In this way, the atom-level graph encodes molecular connectivity, spatial proximity, and local biochemical interaction patterns.

#### 2.4.2 Residue-level subgraph

As a complement to the atom-level graph, we construct a residue-level graph to capture higher-level structural organization of the complex. Protein residue and RNA nucleotide nodes integrate pretrained sequence embeddings with coarse-grained structural features, such as torsion angles and backbone geometry. Edges are introduced between residue or nucleotide nodes whose spatial distance is less than 8 Å. This representation provides an abstract residue-scale description of the complex, complementing the fine-grained biochemical and atomic interaction patterns captured by the atom-level graph and improving tolerance to local conformational variations.

#### 2.4.3 Tri-view structural images

For each protein–RNA complex, three orthogonal views were generated in PyMOL (Schrödinger and DeLano 2023). Each structure was rendered in its original Cartesian coordinate frame after camera resetting and zooming to the visible atoms. The front, side, and top views were obtained using fixed rotations of 0°, 90° about the *y*-axis, and 90° about the *x*-axis, respectively. Protein and RNA chains are color-coded, with distinct colors for individual chains in multi-chain systems to facilitate visual separation. For complexes with multiple experimentally resolved structures available in the PDB, tri-view images were generated independently for each structure using the same standardized workflow.

### 2.5 Overview

**Figure 1:**
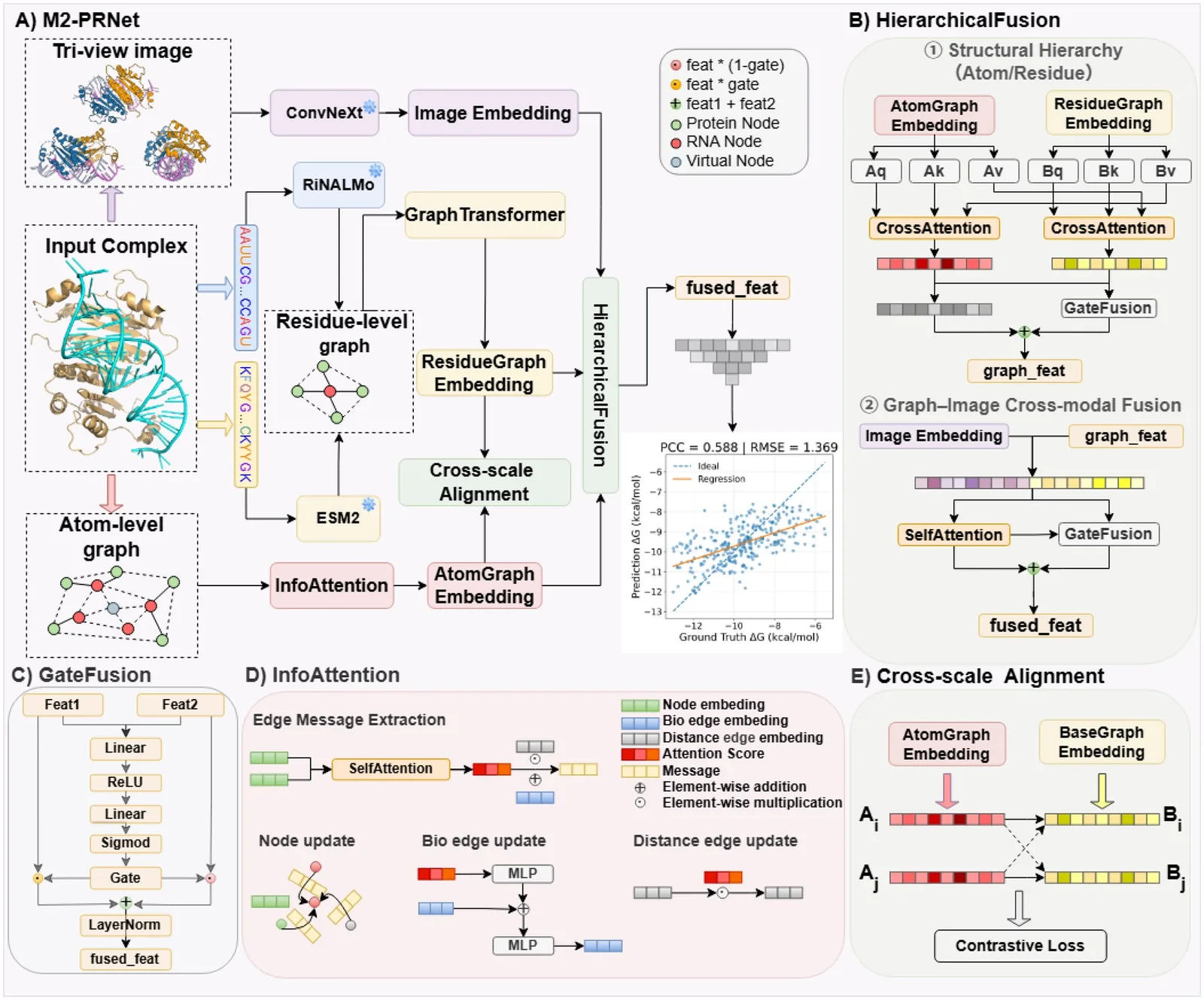
M2-PRNet is a multi-scale and multi-modal framework for protein–RNA binding affinity prediction, jointly leveraging atom-level graphs, residue-level graphs, and tri-view structural images. Atom-level graphs are encoded with an information-aware attention (InfoAttention) mechanism to capture fine-grained atomic interactions, while residue-level graphs are processed by Graph Transformer encoders to model higher-level structural semantics, with cross-scale representations aligned via contrastive learning. Tri-view structural images are encoded using a ConvNeXt backbone to provide complementary global geometric cues. A hierarchical fusion module integrates multi-scale structural and image representations to predict the binding free energy (ΔG).

We propose M2-PRNet, a multi-scale and multi-modal neural framework for protein–RNA binding affinity prediction. As illustrated in Fig. 1, M2-PRNet processes an input RNA–protein complex through three complementary representations: an atom-level interaction graph, a residue-level graph, and tri-view molecular images rendered from the 3D structure.

Each modality is encoded by a dedicated encoder—GNNs for atom- and residue-level graphs and a ConvNeXt backbone for tri-view images—to capture structural, sequential, and spatial cues at different resolutions, followed by a hierarchical fusion module for final affinity prediction.

#### 2.5.1 GateFusion module

To adaptively fuse two heterogeneous feature representations, we introduce a gated fusion module. Given two input features *X* and *Y* of the same dimensionality, a learnable gating function produces an element-wise gate vector $g\in {\left[0,1\right]}^{d}$:


g=σ(G(X,Y)),

where G(·) denotes a compact neural function implemented with a few layers.

The fused representation is computed as


Z=g⊙X+(1−g)⊙Y.

A layer normalization is applied to the fused feature to stabilize optimization.

This formulation enables the model to dynamically balance contributions from different modalities or structural scales.

#### 2.5.2 Hierarchical fusion module

To effectively integrate multi-scale and multi-modal representations, we propose a hierarchical fusion module that progressively fuses atom-level graph features, residue-level graph features, and tri-view image features. Here, “hierarchical” refers to feature integration across different representation scales.

##### 2.5.2.1 Graph–graph fusion

Given atom-level features $Z^{\text{atom}}$ and residue-level features $Z^{\text{res}}$, we first project both to the same latent dimension via linear layers. We then apply bi-directional cross-attention (Vaswani *et al.* 2017) to capture complementary information across structural scales:


$$\begin{array}{c} {\tilde{Z}}^{\text{atom}}=\text{CrossAttn}(Z^{\text{atom}},Z^{\text{res}}),\\ {\tilde{Z}}^{\text{res}}=\text{CrossAttn}(Z^{\text{res}},Z^{\text{atom}}), \end{array}$$

where atom-level features attend to residue-level features and vice versa.

The cross-attended representations are first fused using a GateFusion module:


$$Z^{\text{gf}}=\text{GateFusion}({\tilde{Z}}^{\text{atom}},{\tilde{Z}}^{\text{res}}).$$

To preserve the original projected information, a residual connection from the concatenated and linearly projected features


$$\hat{Z}=\text{Linear}([{\tilde{Z}}^{\text{atom}}\|{\tilde{Z}}^{\text{res}}])$$

is added to the GateFusion output, yielding the final fused graph representation:


$$Z^{\text{graph}}=Z^{\text{gf}}+\hat{Z}.$$

This design enables complementary information exchange between atom- and residue-level representations while preserving their original characteristics through the residual connection.

##### 2.5.2.2 Graph–image fusion

The fused graph features $Z^{\text{graph}}$ are first concatenated with the tri-view image features $Z^{\text{img}}$ extracted by the ConvNeXt backbone to form a joint representation $Z^{\text{con}}$. Self-attention is applied across the concatenated features to capture global dependencies between graph and image modalities, producing $Z^{\text{attn}}$.

A second GateFusion module further integrates $Z^{\text{con}}$ and $Z^{\text{attn}}$, with a residual connection from $Z^{\text{attn}}$, producing the final fused feature representation:


$$Z^{\text{fusion}}=\text{GateFusion}(Z^{\text{con}},Z^{\text{attn}})+Z^{\text{attn}}.$$

The resulting multi-modal representation $Z^{\text{fusion}}$ is fed into a downstream MLP for final protein–RNA affinity prediction. This hierarchical design enables progressive information exchange across structural scales and modalities, producing robust multi-modal representations for affinity prediction.

#### 2.5.3 Atom-level InfoAttention

To capture heterogeneous atomic interactions in protein–RNA complexes, we design an *InfoAttention* mechanism that explicitly models both spatial proximity and biochemical priors through two edge types: distance edges $E_{\text{distance}}$ and biochemical edges $E_{\text{biochemical}}$.

Node features are first projected into query, key, and value representations:


$$Q_{i}=W_{Q}h_{i},\;K_{i}=W_{K}h_{i},\;V_{i}=W_{V}h_{i}.$$

For distance edges, the attention score is modulated by a learnable distance-aware gating function:


$$s_{ij}^{\text{distance}}=\frac{Q_{i}K_{j}^{⊤}}{\sqrt{d}}\cdot e_{ij}^{\text{distance}},$$

where


$$e_{ij}^{\text{distance}}=\text{MLP}(\|x_{i}-x_{j}\|)$$

learns task-specific spatial attenuation.

For biochemical edges, prior interaction features are directly injected into attention scores:


$$s_{ij}^{\text{bio}}=\frac{Q_{i}K_{j}^{⊤}}{\sqrt{d}}+e_{ij}^{\text{bio}},$$

where $e_{ij}^{\text{bio}}$ encodes bond type, stereochemistry, and conjugation information.

The final attention score is selected according to edge type:


$$s_{ij}=\{\begin{array}{cc} s_{ij}^{\text{distance}}, & (i,j)\in E_{\;\text{distance}},\\ s_{ij}^{\text{bio}}, & (i,j)\in E_{\text{biochemical}}. \end{array}$$

Attention weights are normalized over the neighborhood N(i):


$$\alpha_{ij}=\frac{\;\text{exp}(s_{ij})}{\sum_{k\in N(i)}\;\text{exp}\;(s_{ik})}.$$

Node features are then updated via residual message passing:


$$\begin{array}{l} m_{i}=\sum_{j\in N(i)}\alpha_{ij}V_{j},\\ h_{i}^{\text{new}}=(h_{i}+m_{i})+\text{FFN}(h_{i}+m_{i}). \end{array}$$

The edge features are updated in parallel according to their types:


$$\begin{array}{c} e_{ij}^{\text{distance},\text{new}}=(1+\alpha_{ij})⊙e_{ij}^{\text{distance}},\\ e_{ij}^{\text{bio},\text{new}}=e_{ij}^{\text{bio}}+\text{MLP}(\alpha_{ij}). \end{array}$$

#### 2.5.4 Contrastive learning and training objective

To encourage cross-scale consistency, we apply a batch-wise contrastive objective that aligns atom-level and residue-level graph representations of the same protein–RNA complex (Fig. 1d).

Given a mini-batch of *N* complexes, embeddings from the two graph views are compared via a similarity matrix $S\in R^{N\times N}$, where diagonal entries correspond to positive pairs (same complex) and off-diagonal entries serve as negative pairs (different complexes). The contrastive loss is computed using an InfoNCE-style cross-entropy:


$$L_{\text{ctr}}=\text{CE}(S),$$

encouraging atom- and residue-level representations to be aligned.

This contrastive objective is jointly optimized with affinity regression:


$$L=\alpha \cdot \text{MSE}(\hat{y},y)+(1-\alpha )\cdot L_{\text{ctr}},$$

where α=0.2 empirically balances the two objectives, emphasizing predictive accuracy while promoting structurally consistent representations across scales.

## 3 Results

### 3.1 Datasets and experimental setup

#### 3.1.1 Benchmark datasets

Existing protein–RNA binding affinity datasets are limited in size and often contain inconsistent annotations. Following the benchmark construction in CoPRA (Han *et al.* 2025), we integrated complexes from PDBbind (Wang *et al.* 2005), PRBABv2 (Hong *et al.* 2023), and ProNAB (Harini *et al.* 2022), removed duplicates, and reconciled affinity values to obtain 310 unique protein–RNA pairs (**PRA310**). To capture structural variability, we included multiple experimentally resolved conformations for each pair, resulting in 14,113D structures.

To prevent conformation-level information leakage, fold assignment was performed before expanding each complex into multiple structural conformations. All experimentally resolved conformations associated with the same PDB entry inherited the same fold assignment and were never divided between training and test sets. Sequence clustering was also performed before fold construction, so that PDB entries assigned to the same sequence cluster, including entries with identical protein and RNA sequences, were retained in the same fold. Unless otherwise specified, all data splits in this study follow this PDBID grouping principle.

Sequences were clustered with CD-HIT (Fu *et al.* 2012) at 70% identity, and clusters were divided into five folds for cross-validation. A more restrictive subset, **PRA201**, contains only single-chain protein–RNA complexes with additional length constraints. The corresponding PDB IDs for both PRA310 and PRA201 are provided in Table 1, available as supplementary data at *Bioinformatics* online.

**Table 1:** Mean performance of five-fold cross-validation on PRA310 and PRA201.

Method	Input			PRA310				PRA201			
	Structure	Sequence	View	RMSE ↓	MAE ↓	PCC ↑	SPCC ↑	RMSE ↓	MAE ↓	PCC ↑	SPCC ↑
DeePNAP * Pandey *et al.* (2024)	×	✓	×	–	–	–	–	1.964	1.600	0.345	0.349
PredPRBA * Deng *et al.* (2019)	✓	×	×	–	–	–	–	2.238	1.695	0.370	0.316
FoldX ^†^ Buß *et al.* (2018)	✓	×	×	–	–	0.212	0.283	–	–	0.212	0.268
GCN Zhang *et al.* (2019)	✓	×	×	1.705	1.378	0.145	0.144	1.631	1.322	0.201	0.203
GAT Veličković *et al.* (2017)	✓	×	×	1.644	1.337	0.238	0.174	1.542	1.235	0.262	0.221
EGNN Liu *et al.* (2023)	✓	×	×	1.634	1.340	0.226	0.212	1.639	1.345	0.241	0.217
GVP Jing *et al.* (2020)	✓	×	×	1.678	1.361	0.262	0.283	1.702	1.372	0.240	0.305
IPA Jumper *et al.* (2021)	✓	×	×	1.462	1.208	0.495	0.496	1.464	1.191	0.510	0.514
CoPRA (no pretain) Han *et al.* (2025)	✓	✓	×	1.446	1.188	0.522	0.520	1.428	1.172	0.534	0.526
CoPRA Han *et al.* (2025)	✓	✓	×	1.391	1.129	0.580	0.589	1.339	1.059	0.569	0.587
M2-PRNet	✓	✓	✓	**1.368**	**1.078**	**0.601**	**0.612**	**1.301**	**1.037**	**0.598**	**0.627**

↓ indicates lower is better and ↑ indicates higher is better.

* Methods only provide web servers and are evaluated on PRA201 only. FoldX^†^ predicts energy change, thus only correlation is reported. View denotes tri-view structural representations rendered by PyMOL and encoded by a ConvNeXt-Base backbone. Bold values indicate the best performance among all compared methods.

#### 3.1.2 MD-derived dynamic dataset

To examine model behavior on structures that deviate from static experimental conformations, we constructed MD-based evaluation sets following the CoPRA setup (Han *et al.* 2025). After excluding complexes that could not be simulated using standard classical force fields or exceeded feasible system sizes, 30 samples were randomly selected from the test split of each fold, yielding 150 independent protein–RNA systems. Each system was simulated for 1 ns using the *amber99sb-ildn* force field (Lindorff-Larsen *et al.* 2010) and *GROMACS* (Lemkul 2024). We denote the original experimental structures as **MD150^†^** and the corresponding 1 ns MD-sampled structures as **MD150-1ns**.

We further constructed an extended **MD75-10ns** subset to evaluate stronger MD-derived conformational variations. Due to the high computational cost of 10 ns simulations for all 150 complexes, especially larger systems, 75 complexes were selected from MD150 while preserving coverage across different complex-size ranges. For these 75 systems, the original experimental structures, 1 ns MD-sampled structures, and 10 ns MD-sampled structures are denoted as **MD75**, **MD75-1ns**, and **MD75-10ns**, respectively. The PDB IDs of MD150 are listed in Table 2, available as supplementary data at *Bioinformatics* online, and the complex lengths and corresponding ranks of the selected MD75-10ns complexes are provided in Table 3, available as supplementary data at *Bioinformatics* online. All MD-derived datasets were used exclusively for evaluation and were not included in model training.

**Table 2:** Performance comparison of structure-based methods on MD150^†^ and MD150-1ns.

Dataset	Method	RMSE ↓	MAE ↓	PCC ↑	SPCC ↑
MD150^†^	GVP	1.184	0.952	0.330	0.247
MD150^†^	IPA	1.089	0.866	0.493	0.455
MD150^†^	CoPRA	1.068	0.851	0.529	0.468
MD150^†^	M2-PRNet	**0.931**	**0.744**	**0.592**	**0.578**
MD150-1ns	GVP	1.187	0.946	0.309	0.234
MD150-1ns	IPA	1.192	0.938	0.353	0.339
MD150-1ns	CoPRA	1.059	0.847	0.541	0.478
MD150-1ns	M2-PRNet	**0.823**	**0.717**	**0.657**	**0.524**

MD150^†^ denotes the original experimental structures contained in PRA310, while MD150-1ns denotes the corresponding 1 ns MD-sampled structures. Bold values indicate the best performance among all compared methods.

#### 3.1.3 Experimental setup

Protein and RNA sequence representations are extracted using ESM-2 (650M) and RiNALMo (650M), respectively, while tri-view structural features are extracted using an ImageNet-pretrained ConvNeXt-Base backbone. Following (Han *et al.* 2025), we evaluate all models using RMSE, MAE, PCC, and SPCC. Models are trained on a single NVIDIA L20 GPU for approximately 20 hours using AdamW with a learning rate of $1\times 10^{-4}$. Following the same PDB ID-level grouping principle, 10% of PDB IDs from each training fold are selected for validation, and all corresponding structures are assigned to the validation set. Because different PDB IDs contain different numbers of available structures and balanced sampling changes the number of sampled instances per epoch, validation is performed every 100 steps rather than once per epoch. Early stopping is triggered after 20 evaluations without improvement. Detailed parameter and hyperparameter settings are provided in Tables 4 and 5, available as supplementary data at *Bioinformatics* online.

### 3.2 Evaluation on benchmark datasets

We evaluate M2-PRNet on the PRA310 and PRA201 benchmark datasets and compare it with a broad range of baselines. PRA310 contains structurally complex multi-chain protein–RNA assemblies, whereas PRA201 mainly consists of simpler single-chain complexes. As shown in Table 1, M2-PRNet achieves the best performance across all metrics, reaching an RMSE of 1.368 and a PCC of 0.601 on PRA310, and an RMSE of 1.301 and a PCC of 0.598 on PRA201.

M2-PRNet consistently outperforms both the non-pretrained and pretrained variants of CoPRA, demonstrating the effectiveness of integrating complementary multi-level structural representations with pretrained sequence and image features. The performance gains are particularly pronounced on PRA310, highlighting the benefit of integrating atom-level interactions, residue-level organization, and global structural context for challenging, multi-chain complexes. On PRA201, M2-PRNet still achieves consistent improvements, demonstrating robust generalization across different structural regimes.

To further examine the prediction behavior, we provide scatter plots of predicted versus experimental affinities in Fig. 4. The results show that compared with GVP, IPA, and CoPRA, M2-PRNet produces predictions that are more closely aligned with the regression line, achieving a higher coefficient of determination $R^{2}$.

**Figure 2:**
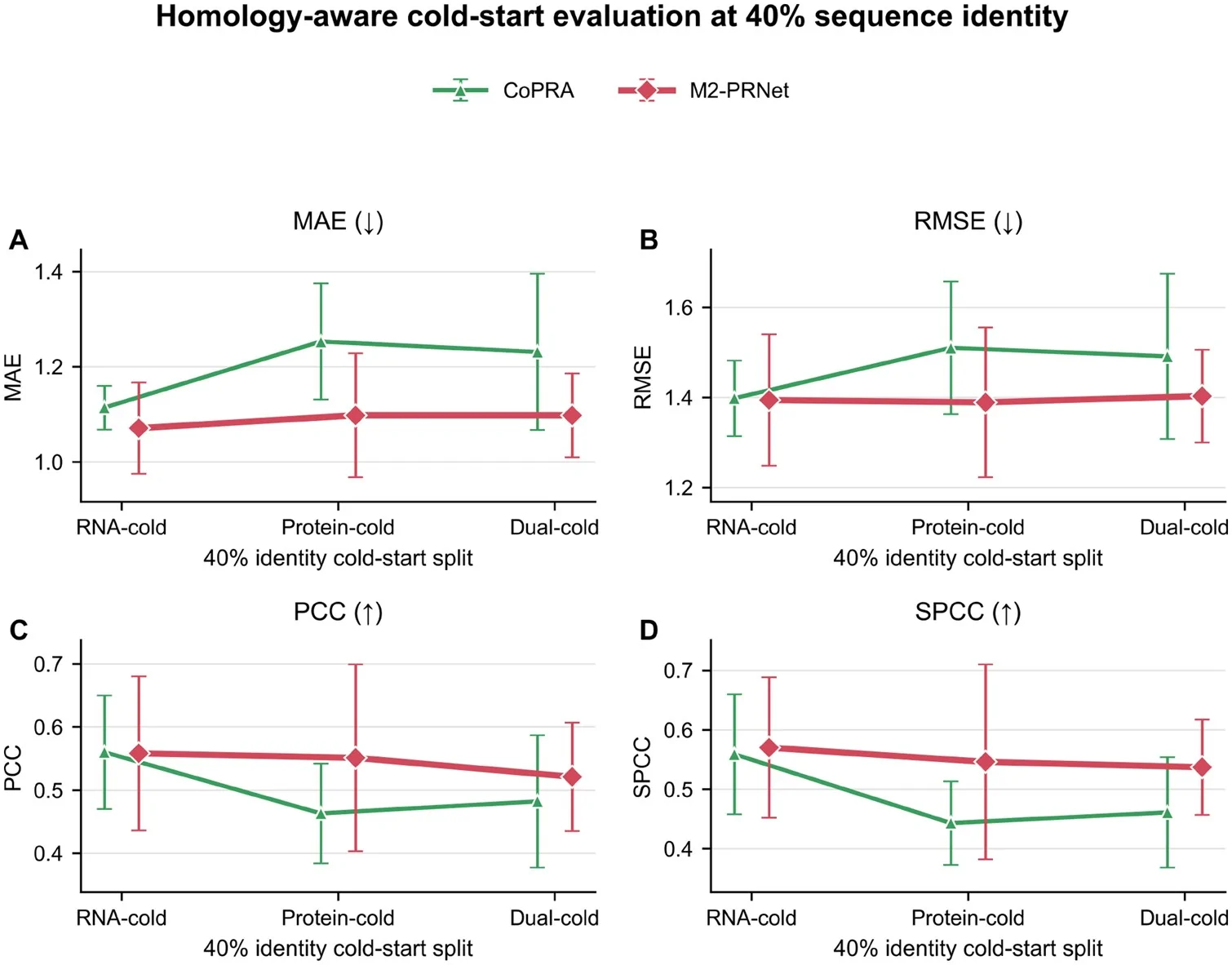
Scatter plots of predicted versus experimental binding affinities on the PRA310 results for different models.

Overall, these results confirm the effectiveness and robustness of M2-PRNet for protein–RNA binding affinity prediction.

### 3.3 Homology-aware cold-start evaluation

To provide a more rigorous evaluation of cold-start generalization, we constructed a homology-aware benchmark using a 40% sequence identity threshold. RNA sequence clustering was performed using MMseqs2 (Steinegger and Söding 2017), and protein sequence homology was assessed using BLASTP (Camacho *et al.* 2009). Three cold-start settings were considered. In the RNA-cold split, RNAs in the test set shared less than 40% sequence identity with RNAs in the training set. In the protein-cold split, proteins in the test set shared less than 40% sequence identity with training proteins. In the dual-cold split, both RNA and protein sequences in the test set satisfy a sequence identity threshold of less than 40% relative to the training set.

As shown in Fig. 2 and Table 6 available as supplementary data at Bioinformatics online, M2-PRNet achieved performance comparable to CoPRA in the RNA-cold setting, with slightly lower MAE and slightly higher SPCC. More evident improvements were observed in the protein-cold and dual-cold settings. In the protein-cold split, M2-PRNet reduced MAE and RMSE and improved both PCC and SPCC compared with CoPRA. In the dual-cold split, M2-PRNet also achieved lower prediction errors and higher rank correlation. Compared with the original 70% identity split, this 40% homology-aware benchmark provides a more conservative assessment of cross-complex generalization. These results indicate that M2-PRNet maintains competitive generalization performance for less-homologous protein targets and for jointly less-homologous RNA–protein pairs.

**Figure 3:**
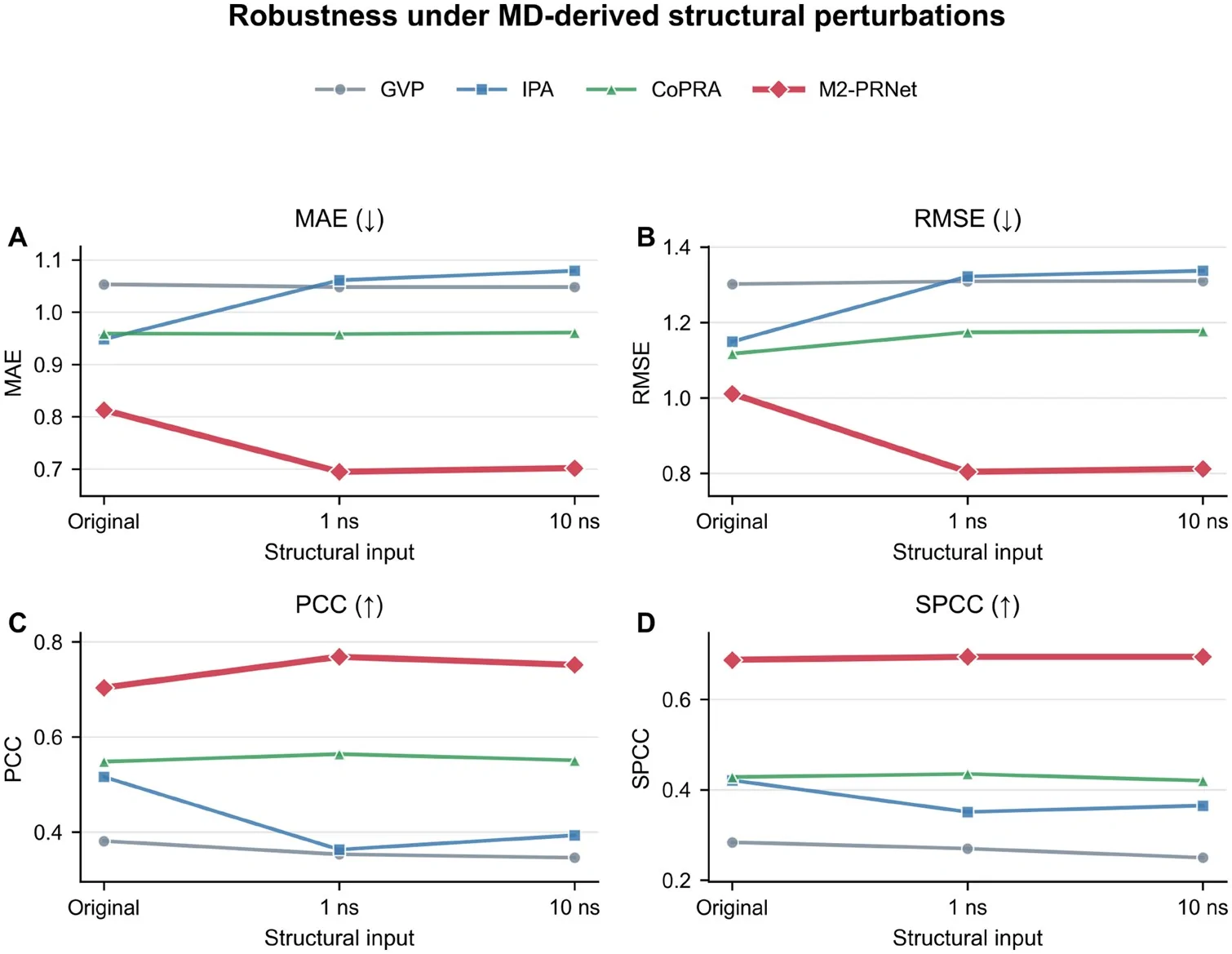
Homology-aware cold-start evaluation under a 40% sequence identity threshold. CoPRA and M2-PRNet were evaluated under RNA-cold, protein-cold, and dual-cold settings using (a) MAE, (b) RMSE, (c) PCC, and (d) SPCC. Points and error bars represent the mean and standard deviation, respectively.

A limitation of the present cold-start evaluation is that the training–test split was constructed primarily according to sequence similarity. Low sequence similarity does not necessarily imply structural novelty, because proteins with divergent sequences may still adopt similar folds or belong to the same structural superfamily. To further assess this issue, we performed an additional SCOPe-based (Chandonia *et al.* 2019) analysis for the PRA310 dataset. Due to the limited coverage of SCOPe annotations, 102 out of 310 protein components could be mapped to SCOPe domains. For these annotated samples, we report the family-, superfamily-, and fold-level overlap between the training and test sets in Table 7, available as supplementary data at *Bioinformatics* online. The analysis shows that although our split removes identical PDB entries and controls sequence similarity using MMseqs2-based clustering, it does not fully eliminate overlap at the structural-domain or fold level. Thus, the current results primarily demonstrate generalization under a sequence-based split and should not be interpreted as conclusive evidence of generalization to entirely unseen protein folds. Structure-aware splitting and evaluation, for example based on SCOPe classifications or direct structural similarity measures such as TM-score, remain important directions for future work.

### 3.4 Performance on MD-derived dynamic structures

To evaluate robustness to MD-derived conformational fluctuations, we tested M2-PRNet on the MD150-1ns dataset. Since sequence-only methods are largely insensitive to structural perturbations, we compared M2-PRNet with recent structure-based methods, using CoPRA as the strongest baseline. As shown in Table 2, M2-PRNet achieved the best performance on both the original crystal structures (MD150^†^) and the 1 ns MD-sampled structures (MD150-1ns). On MD150^†^, M2-PRNet achieved a PCC of 0.592, compared with 0.529 for CoPRA. On MD150-1ns, it achieved a PCC of 0.657 and an RMSE of 0.823. These results suggest that M2-PRNet maintains strong predictive performance under short-timescale, MD-derived local structural perturbations.

To further investigate robustness under more pronounced MD-derived conformational variations, we evaluated M2-PRNet on the previously defined MD75-10ns subset. We first quantified the structural variation introduced by the extended simulations. Compared with the corresponding 1 ns trajectories, the 10 ns simulations produced clearly larger RMSD and RMSF values, indicating that MD75-10ns represents a stronger structural perturbation setting. Therefore, this subset provides an additional evaluation under larger, but still moderate, MD-derived structural deviations. As shown in Table 8, available as supplementary data at *Bioinformatics* online, the RMSD increased from 0.837 ± 0.100 Å for MD75-1ns to 1.784 ± 0.505 Å for MD75-10ns, and the RMSF increased from 0.505 ± 0.053 Å to 1.048 ± 0.326 Å.

Detailed numerical results for MD75, MD75-1ns, and MD75-10ns are provided in Table 9, available as supplementary data at *Bioinformatics* online. As shown in Fig. 3, M2-PRNet achieved the most favorable mean performance among the compared structure-based methods across the three evaluated settings. In contrast, GVP and IPA showed relatively limited performance under both 1 ns and 10 ns MD-derived structures, while CoPRA remained stable but did not reach the accuracy of M2-PRNet. Notably, both CoPRA and M2-PRNet maintained stable performance from MD75-1ns to MD75-10ns, while M2-PRNet consistently outperformed the compared structure-based methods across all three MD-derived settings. Together with the RMSD and RMSF results, this suggests that the 10 ns trajectories introduced stronger structural deviations than the 1 ns trajectories, while still remaining within a moderate perturbation range. Therefore, the MD75-10ns experiment mainly evaluates robustness to local-to-moderate MD-derived conformational variations, and M2-PRNet showed stable performance under this setting.

**Figure 4:**
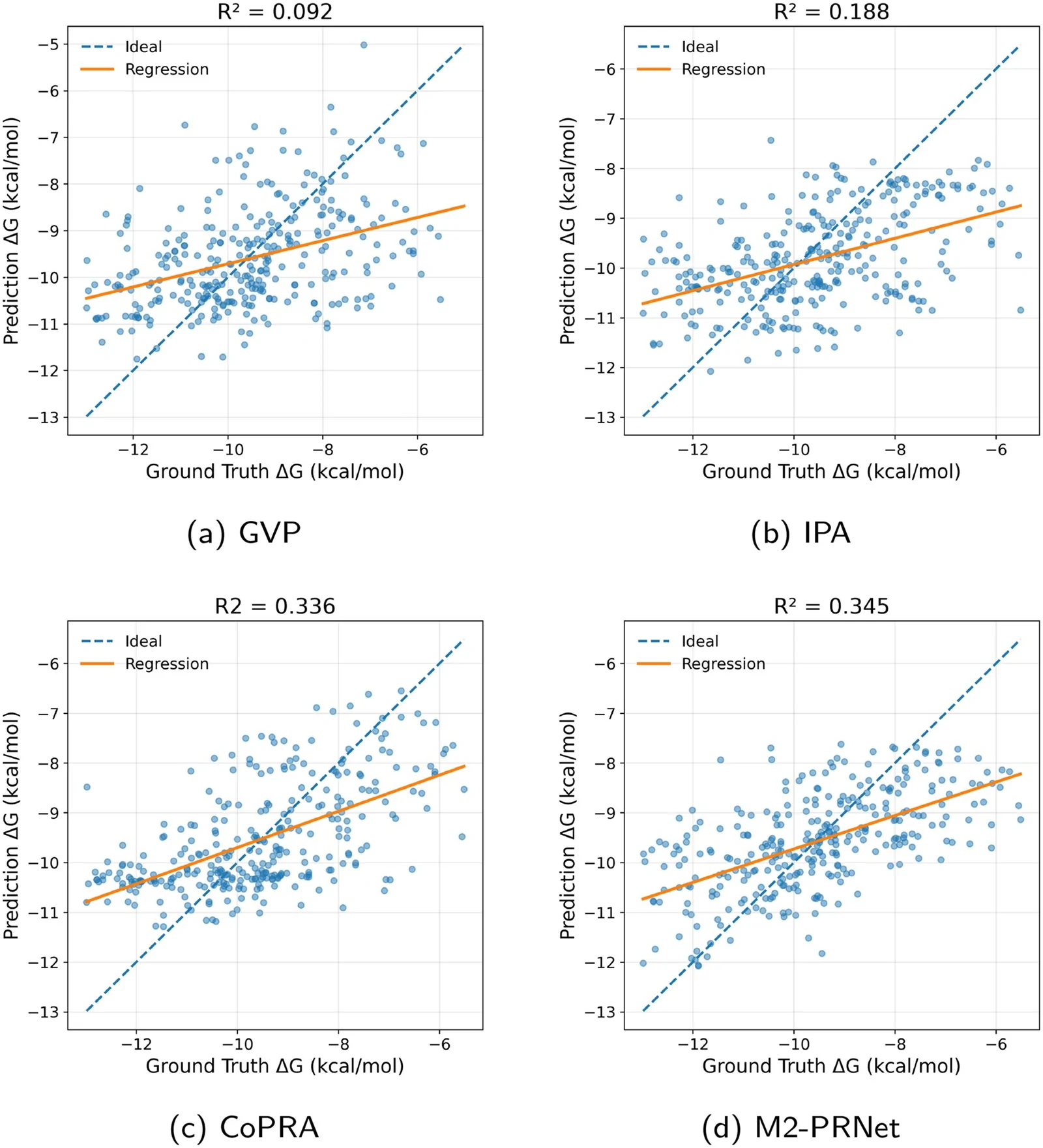
Performance comparison on MD-derived protein–RNA complex structures. The performance of GVP, IPA, CoPRA, and M2-PRNet was evaluated on MD75, MD75-1ns, and MD75-10ns using (a) MAE, (b) RMSE, (c) PCC, and (d) SPCC.

Overall, the MD75-10ns experiment provides a more challenging MD-derived perturbation setting than the original 1 ns MD evaluation, with structural deviations mainly corresponding to local-to-moderate conformational variations. The stable performance of M2-PRNet under this setting suggests its ability to tolerate moderate MD-derived structural perturbations. This robustness may benefit from the use of all experimentally available PDB structures for the same protein–RNA pair during training, which exposes the model to experimentally observed conformational diversity, as well as from the integration of atom-level, residue-level, and global structural representations.

### 3.5 Case studies

#### 3.5.1 Attention-based visualization of representative complexes

We further evaluated model interpretability using three representative case studies: 1URN, VEGF165, and 4BS2. 1URN and 4BS2 are based on experimentally resolved PDB structures, whereas VEGF165 is included as a predicted-structure applicability case using an AlphaFold3-predicted complex model. Therefore, these examples are used as qualitative case studies rather than as evidence for rigorous cold-start generalization.

**Figure 5:**
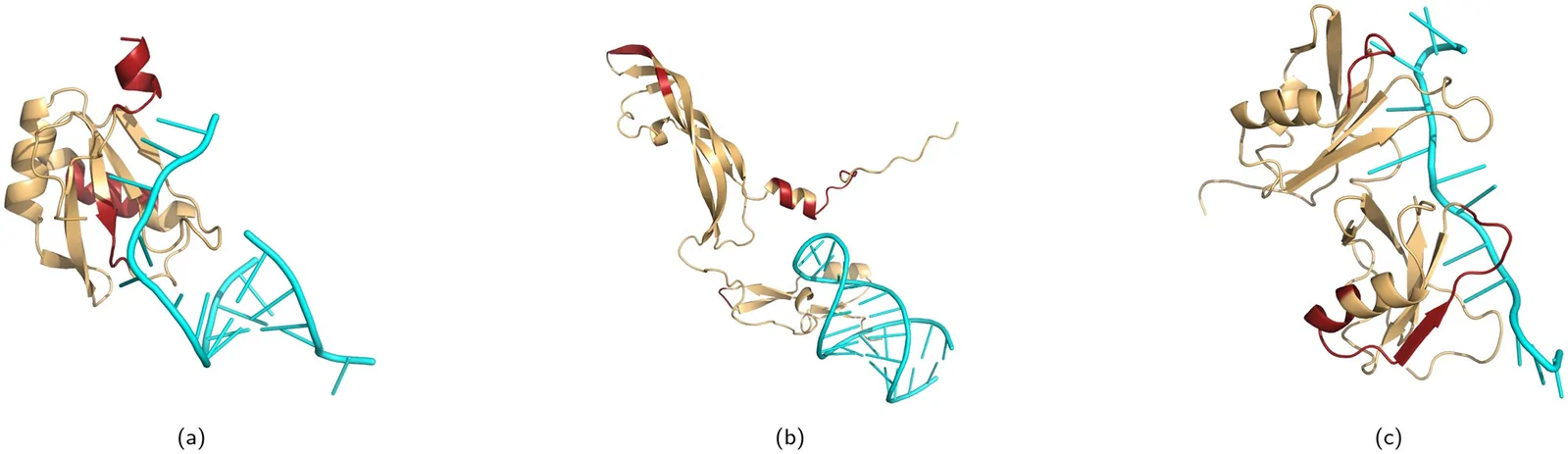
Visualization of three representative protein–RNA complexes: (a) 1URN, (b) VEGF165, and (c) 4BS2. Proteins and RNA are shown in distinct colors; the top 20 residues with the highest attention weights are highlighted in red.

As shown in Fig. 5, proteins are colored light orange, RNA cyan, and the top 20 attention-ranked residues are highlighted in red. For 1URN, a canonical RNA recognition motif, high-attention residues are mainly located on the β-sheet surface and nearby α-helices, consistent with its known RNA-binding interface (Maris *et al.* 2005). For VEGF165, the protein target of the RNA aptamer drug Pegaptanib, attention is more dispersed, possibly reflecting uncertainty associated with the AlphaFold3-predicted complex structure. Nevertheless, some high-attention residues are located near the predicted aptamer-binding region, suggesting that the model can still provide qualitative interface-level signals when predicted structures are used (Katz and Goldbaum 2006). For 4BS2, high-attention residues span multiple domains and form a continuous interface along the RNA, suggesting cooperative RNA recognition (Lunde *et al.* 2007).

Overall, these case studies suggest that M2-PRNet can highlight functionally relevant RNA-binding regions in experimentally resolved complexes. The VEGF165 case further illustrates a predicted-structure application scenario, where the interpretation should be treated as qualitative and depends on the quality of the generated complex structure.

##### 3.5.2 Evaluation on predicted protein–RNA structures

To evaluate the applicability of M2-PRNet to predicted protein–RNA complex structures, we analyzed three VEGF165-binding aptamers and a scrambled RNA control (Ruckman *et al.* 1998) using AlphaFold3-generated complexes. As shown in Table 10, available as supplementary data at *Bioinformatics* online, M2-PRNet assigned stronger mean predicted affinities to the three functional aptamers than to the scrambled control, although the fine-grained ranking among the three aptamers was not fully recovered. Interface-level analysis showed a similar trend: RNA interface coverage decreased from 70.4% for t44-OMe to 65.2% for t22-OMe, 57.2% for t2-OMe, and 46.7% for scr-t44, and the functional aptamers formed larger predicted interfaces (1118–1165 Å²) than the scrambled control (773 Å²). These results suggest that M2-PRNet may assist with preliminary discrimination between strong and weak binders when plausible predicted structures are available. However, the relatively low interface confidence scores and the absence of explicit chemical modifications in the predicted structures indicate that absolute affinity prediction and fine-grained ranking remain dependent on input-structure quality.

### 3.6 Ablation studies

#### 3.6.1 Effect of multi-level structural representations

We evaluated the contributions of the atom-level graph, residue-level graph, tri-view image branch, and contrastive learning objective. As summarized in Table 11, available as supplementary data at *Bioinformatics* online, removing any component consistently degrades performance on both PRA310 and PRA201. The residue-level graph produces the largest contribution on PRA310, while the atom-level graph and tri-view image branch provide complementary fine-grained interaction and global structural information, respectively.

Figure 1, available as supplementary data at *Bioinformatics* online, further shows that covalent bond edges, IFP-based interaction edges, distance-based edges, and virtual aromatic nodes all have non-zero learned weight distributions, supporting their participation in atom-level message passing. In addition, the t-SNE visualization in Fig. 2, available as supplementary data at *Bioinformatics* online, shows that fused representations achieve clearer affinity-level separation than individual modality-specific features, indicating the benefit of multi-level structural fusion.

#### 3.6.2 Effect of training strategies

To further investigate the contribution of different components, we conducted ablation studies on contrastive learning and multi-modal fusion (Table 11, available as supplementary data at *Bioinformatics* online). Removing either module leads to noticeable performance degradation, confirming their importance for accurate affinity prediction. In particular, the **-fusion** variant shows the largest drop, highlighting the benefit of integrating multi-level structural information.

The **+tri-con** variant also underperforms compared with the standard contrastive design, suggesting that directly aligning all modalities may introduce optimization difficulty due to their heterogeneity.

We further analyzed the impact of tri-view image resolution and observed that higher resolutions yield diminishing performance gains while increasing computational cost. Therefore, 1200 was selected as a balanced choice (Fig. 3, available as supplementary data at *Bioinformatics* online).

## 4 Conclusion

We propose M2-PRNet, a multi-scale and multi-modal framework for protein–RNA binding affinity prediction. By integrating atom-level graphs, residue-level graphs, and tri-view molecular images, M2-PRNet captures fine-grained biochemical interactions, residue-scale structural semantics, and global binding geometry. A cross-scale contrastive objective further aligns atom- and residue-level representations across different structural resolutions.

Experiments on PRA310 and PRA201 demonstrate state-of-the-art performance under clustering-based five-fold cross-validation. Under stricter homology-aware RNA-cold, protein-cold, and dual-cold splits with a 40% sequence identity threshold, M2-PRNet maintains competitive performance, supporting its generalization under reduced sequence homology. In MD-derived evaluations, including MD150-1ns and the extended MD75-10ns subset, the model remains stable under local-to-moderate structural perturbations, with MD75-10ns showing larger RMSD and RMSF values than the corresponding 1 ns trajectories.

Ablation and case studies further support the contributions of multi-level structural representations, contrastive learning, and gated fusion. M2-PRNet can highlight relevant RNA-binding regions in representative complexes and, in a VEGF165 predicted-structure case, distinguish high-affinity aptamers from a low-affinity scrambled control using AlphaFold3-generated complexes. Overall, these results demonstrate that integrating multi-scale structural representations and multi-modal fusion provides an effective framework for protein–RNA affinity prediction. By enabling structure-based affinity assessment, interface interpretation, and preliminary discrimination between strong and weak RNA binders, M2-PRNet may serve as an auxiliary computational tool for RNA-targeted drug discovery when plausible complex structures are available.

## Supplementary Material

btag614_Supplementary_Data

## Data Availability

All data used in this study are available at https://github.com/CSUBioGroup/M2-PRNet.
