# Supplementary material for: M2-PRNet: multi-scale and multi-modal learning for protein–RNA binding affinity prediction
Source: Bioinformatics. 2026 Aug 17;42(8):btag614. doi: 10.1093/bioinformatics/btag614 (PMC13516915; doi:10.1093/bioinformatics/btag614)
Supplement: Supplementary file 1 [file btag614_supplementary_data.pdf]

Table S1 PDB IDs for the PRA310 and PRA201 datasets.

Dataset	PDBID
PRA310	6DCC,3NMR,6RT6,2MJH,2HGH,2I91,1RPU,3QG9,2KG0,1YTU,3Q0N,3R2C,2GJE,3Q0M,3K61,2MFF,3V7E,1EC6,5WWF,6FPX,4PDB,4ERD,2KXN,5ZC9,6O16,2A9X,4RCJ,4KJI,6G99,2MQV,3V74,4N2Q,4Z4D,2ZZN,1NYB,4OOG,3AEV,3TRZ,5ID6,4Z4E,5WZG,5U9B,6NOC,4QQB,6DB9,4L8R,1RLG,1ASY,4B3G,6RT7,1Y39,2LA5,6NOH,6NY5,1U0B,6GX6,4R3I,5F9F,2LUP,3AM1,3ADL,3UZS,4Z4C,2MFG,3PF5,4QVD,3V6Y,5YTV,5YTS,4LCK,3IAB,4JXX,1WWG,3V71,4I67,4N2S,1ASZ,6DCB,3BX3,1RKJ,1S03,2ZI0,4C7O,5E3H,6DCL,2IHX,4JVY,3RW6,6C6K,6G2K,3BSU,1T0K,1KOG,4Z4F,2MFH,4CIO,3K4E,3MDG,3EQT,1YTY,3Q0S,5Y58,5H1L,2L2K,5WWW,2BU1,6B3K,5MPL,2LBS,1N77,1N78,6NOF,5SZE,2LEB,4NGB,3BSX,4Z7K,6SDW,6CMN,6MCE,2IX1,5DET,1F6U,6KWR,3I5X,4Z4H,2QUX,6KWQ,4JVH,5HO4,2RRA,5ZKJ,2LEC,3NCU,3ADB,6F4H,2RU3,3K64,4Z4I,3QGC,2M8D,2MFC,3SIU,4Z7L,5WZH,3Q0L,2XS2,4YVI,6MCF,4GHL,2RQC,2G4B,2ANN,3K5Y,3R9W,6SO9,6GC5,6FQR,6U8D,3IWN,3K5Z,3DD2,5ELR,1QFQ,4NKU,1M8Y,6PUN,2C4R,4MDX,4OE1,2XZL,1YVP,4KZE,1M8W,3LRN,2UWM,5GXH,5F98,6U9X,6HYU,2Y8W,5JBJ,1U63,2KG1,6FQ3,3EGZ,5WLH,4QI2,1WWE,2L41,6SDY,5TF6,5GXI,1A4T,2AZ0,2VPL,5E08,3Q0P,1WWD,4QG3,4QVC,4C9D,3BX2,2XC7,5EIM,2LI8,3K0J,3O3I,5YTT,1ZH5,1MMS,5ZTM,5YKI,1KNZ,5KLA,6R7B,3O7V,5DNO,5KL1,6NOD,5UDZ,2MB0,4PKD,2XFM,2YH1,2PJP,2R8S,3FHT,6A6J,5M8I,3O6E,4TUX,2ZKO,4LJ0,2C4Y,5F5H,6B14,3K5Q,3PF4,2KFY,2KX5,4JYZ,2MKN,4BS2,1WWF,2MFE,3UZT,5W6V,2MKI,2N82,1M8X,1L1C,5KL8,4O26,3K62,5WWG,1K1G,2MTV,2F8K,4ED5,3QGB,5WZJ,5WZK,2MKK,5DV7,2X1A,1QTQ,2MXY,1IL2,1K8W,3K49,1JBR,5JB2,4YHW,2MS1,5EN1,2MZ1,5W1H,4M59,1FJE,1OOA,4QEI,5V7C,1U6P,6F4G,4B8T,6GBM,5W1I,5H1K,3D2S,5F9H,1G59,4Z4G,4JXZ,5WWX,2C4Q,3V11,3VYX,6FPQ,5MPG,1WSU,6D12,1ZHO,2JPP,5YTX,2B6G,5WWE,3BSB,2L3C,4QVI
PRA201	6DCC,3NMR,6RT6,2MJH,2HGH,3QG9,2KG0,3Q0N,3Q0M,3K61,1EC6,5WWF,6FPX,4PDB,2KXN,5ZC9,6O16,2A9X,4RCJ,4KJI,6G99,2MQV,3V74,4N2Q,1NYB,5WZG,5U9B,6NOC,1RLG,4B3G,6RT7,1Y39,2LA5,6NOH,6GX6,4R3I,5F9F,2LUP,3UZS,3PF5,3V6Y,5YTV,5YTS,4JXX,1WWG,3V71,4I67,4N2S,6DCB,3BX3,1RKJ,1S03,4JVY,3RW6,6C6K,4CIO,3K4E,3MDG,1YTY,3Q0S,5H1L,2L2K,5WWW,5MPL,2LBS,6NOF,5SZE,2LEB,4NGB,3BSX,6SDW,6CMN,6MCE,2IX1,5DET,1F6U,3I5X,4JVH,5HO4,2RRA,5ZKJ,2LEC,6F4H,2RU3,3K64,3QGC,2M8D,4Z7L,5WZH,3Q0L,2XS2,6MCF,2RQC,2G4B,2ANN,3K5Y,3R9W,6SO9,6GC5,6FQR,3K5Z,1QFQ,4NKU,1M8Y,6PUN,2C4R,2XZL,5GXH,5F98,6U9X,6HYU,2Y8W,1U63,2KG1,6FQ3,4QI2,1WWE,2L41,6SDY,5TF6,5GXI,1A4T,2VPL,3Q0P,1WWD,4QVC,4C9D,3BX2,2XC7,5EIM,2LI8,3O3I,5YTT,1ZH5,1MMS,5YKI,5KLA,3O7V,5DNO,6NOD,2MB0,4PKD,2XFM,2YH1,2PJP,3FHT,6A6J,5M8I,3O6E,4TUX,5F5H,3K5Q,3PF4,2KFY,2KX5,4JYZ,4BS2,1WWF,3UZT,5W6V,2MKI,2N82,1M8X,4O26,3K62,5WWG,1K1G,2MTV,2F8K,4ED5,3QGB,5WZJ,5WZK,2MKK,2MXY,1K8W,3K49,4YHW,2MS1,5EN1,2MZ1,1FJE,1OOA,4QEI,5V7C,4B8T,6GBM,5H1K,3D2S,5F9H,4JXZ,5WWX,6FPQ,5MPG,1WSU,1ZHO,5YTX,2B6G,5WWE,3BSB,2L3C

Table S2 PDB IDs for the MD150 datasets.

Dataset	PDBID
MD150	2MJH,3R2C,6GX6,3ADL,3V71,1S03,2IHX,4JVY,3RW6,2BU1,6B3K,6MCE,4Z7L,6MCF,6U8D,4KZE,6HYU,5E08,2PJP,2R8S,4TUX,2C4Y,6B14,2F8K,2MXY,2MZ1,2C4Q,3VYX,6D12,2B6G,3NMR,3K61,4ERD,5U9B,6NOC,2LA5,6NOH,4R3I,3UZS,4QVD,3IAB,6C6K,4CIO,6NOF,4JVH,2RU3,3K64,3K5Y,3K5Z,6PUN,6U9X,5UDZ,3K5Q,2KFY,1L1C,3K62,2MTV,2X1A,3D2S,2JPP,6DCC,2GJE,1NYB,5WZG,1RLG,6NY5,3PF5,6DCB,3BX3,6DCL,1T0K,5HO4,5WZH,4MDX,1YVP,2Y8W,5JBJ,2KG1,4QG3,3BX2,6NOD,3FHT,3PF4,4BS2,2N82,4ED5,5WZJ,5WZK,4YHW,1ZHO,2HGH,2KXN,4KJI,4B3G,3AM1,5YTV,5YTS,2ZI0,3EQT,5H1L,4NGB,2IX1,6KWR,6KWQ,2RRA,3ADB,2M8D,4YVI,2RQC,1M8Y,4QI2,5YTT,6A6J,5F5H,2MKN,1M8X,1K8W,4M59,5H1K,5YTX,1YTU,6FPX,4PDB,4L8R,1WWG,4N2S,3K4E,6SDW,1F6U,3NCU,6F4H,3R9W,3IWN,2C4R,3EGZ,6SDY,1A4T,1WWD,3K0J,1KNZ,6R7B,5DNO,4PKD,4LJ0,1WWF,5W6V,2MKI,1K1G,2MS1,6F4G

Table S3. Length distribution and ranking of the selected MD75-10ns complexes. Complexes are ranked in ascending order according to the total complex length. The table shows that the selected systems cover different complex-size ranges.

PDBID	Complex length	rank
1A4T	34	1
1NYB	46	2
2LA5	53	4
1WWG	62	6
1WWD	62	5
1WWF	63	7
3PF4	71	8
3PF5	72	9
1F6U	73	11
3D2S	73	12
2MKN	82	19
2X1A	89	20
3ADL	96	22
2M8D	99	24
2F8K	100	26
2B6G	100	25
2KXN	101	27
2RRA	105	29
2KFY	108	31
2RU3	109	33
2MZ1	110	34
2KG1	111	35
2MXY	112	36
2RQC	121	39
1T0K	124	40
2IHX	125	41

PDBID	Complex length	rank
2MS1	126	43
2N82	127	44
1K1G	133	45
1RLG	135	46
1L1C	139	47
2HGH	142	48
2PJP	144	49
2JPP	146	50
2MJH	152	52
2ZI0	153	53
3EGZ	153	54
2MTV	161	56
3NMR	171	61
1S03	174	64
3IWN	184	67
2MKI	208	73
3R2C	226	75
2Y8W	234	77
3K0J	261	82
3NCU	262	83
1ZHO	263	84
2C4Y	274	87
2C4Q	274	86
2BU1	274	85
3EQT	285	88
3IAB	297	93
1KNZ	297	92
2GJE	299	95
3RW6	305	96
3AM1	317	98
1K8W	325	100
3BX3	333	102
3R9W	336	103
3BX2	337	104
1M8X	349	105
1M8Y	351	106
3K4E	353	108
3FHT	398	112
3K61	402	113
3K5Z	403	114
3K5Y	409	117
3K5Q	409	116
3K62	409	118

PDBID	Complex length	rank
3K64	409	119
1YTU	410	120
2C4R	501	127
1YVP	550	137
3UZS	629	142
2IX1	656	144

Table S4. The hyperparameter configuration of M2-PRNet

Neural network component	Hyperparameters	Value of hyperparameter
InfoAttention	Head num	4
	Attention_dropout_rate	0.2
	Node hidden dimension	128
	Edge hidden dimension	64
	Number of layers	2
	Attention heads	4
	Head dimension	32
GraphTransformer	RNA biochemical feature dim	9
	RNA sequence embedding dim	1280
	Protein biochemical feature dim	8
	Protein sequence embedding dim	1280
	Hidden dimension	256
	Transformer layers	6
	Attention heads	8
ConvNeXt-Base	88.6M Parameters	frozen
ESM-2	650M Parameters	frozen
RiNALMo	650M Parameters	frozen
Overall architecture	Loss function	Mean Squared Error Loss
	Optimizer	AdamW
	Distance threshold	8Å
	Graph network layer depth	2
	Learning rate	0.0001
	MSE weight	0.8
	Contrastive learning weight	0.2
	Dropout rate	0.2
	Batch size	8
	Epochs	8

Table S5. Trainable parameter distribution of M2-PRNet.

Module	Trainable parameters
GraphTransformer	5.11M
Atom-level graph encoder	0.41M
Fusion and prediction module	0.23M

Table S6. Homology-aware cold-start evaluation under a 40% sequence identity threshold.

Method	split	MAE(↓)	RMSE (↓)	PCC (↑)	SPCC (↑)
CoPRA	RNA-cold	1.114±0.046	1.398±0.084	0.560±0.090	0.559±0.101
M2-PRNet	RNA-cold	1.071±0.096	1.394±0.146	0.558±0.122	0.570±0.118
CoPRA	protein-cold	1.253±0.122	1.510±0.147	0.463±0.079	0.443±0.070
M2-PRNet	protein-cold	1.098±0.130	1.389±0.166	0.551±0.148	0.546±0.164
CoPRA	dual-cold	1.231±0.164	1.491±0.183	0.482±0.105	0.461±0.093
M2-PRNet	dual-cold	1.098±0.088	1.403±0.103	0.521±0.086	0.537±0.080

Table S7. SCOPe family-, superfamily-, and fold-level overlap between training and test sets.

Fold	Level	Test samples	SCOPe-annotated test samples	Overlapping test samples	Overlap among annotated test samples (%)	Overlap among all test samples (%)
Fold_0	family	71	19	10	52.63%	14.08%
Fold_0	superfamily	71	19	11	57.89%	15.49%
Fold_0	fold	71	19	13	68.42%	18.31%
Fold_1	family	54	21	10	47.61%	18.51%
Fold_1	superfamily	54	21	10	47.61%	18.51%
Fold_1	fold	54	21	10	47.61%	18.51%
Fold_2	family	58	16	13	81.25%	22.41%
Fold_2	superfamily	58	16	14	87.50%	24.13%
Fold_2	fold	58	16	15	93.75%	25.86%
Fold_3	family	55	17	11	64.71%	20.00%
Fold_3	superfamily	55	17	12	70.59%	21.82%
Fold_3	fold	55	17	14	82.35%	25.45%
Fold_4	family	72	29	14	48.28%	19.44%
Fold_4	superfamily	72	29	24	82.75%	33.33%
Fold_4	fold	72	29	24	82.75%	33.33%

Table S8. RMSD and RMSF statistics of the MD75-1ns and MD75-10ns subsets.

Dataset	RMSD(Å)	RMSF(Å)
MD75-1ns	0.837 ± 0.100	0.505 ± 0.053
MD75-10ns	1.784 ± 0.505	1.048 ± 0.326

Table S9. Performance comparison of different models on MD75, MD75-1ns and MD75-10ns.

Method	Dataset	MAE(↓)	RMSE (↓)	PCC (↑)	SPCC (↑)
GVP	MD75	1.053±0.153	1.302±0.230	0.381±0.271	0.284±0.278
IPA	MD75	0.948±0.284	1.149±0.350	0.516±0.219	0.421±0.177
CoPRA	MD75	0.959±0.276	1.117±0.351	0.548±0.197	0.428±0.197
M2-PRNet	MD75	0.813±0.225	1.011±0.276	0.704±0.041	0.687±0.058
GVP	MD75-1ns	1.048±0.143	1.309±0.234	0.353±0.281	0.270±0.269
IPA	MD75-1ns	1.061±0.297	1.322±0.412	0.363±0.369	0.351±0.301
CoPRA	MD75-1ns	0.958±0.267	1.174±0.353	0.564±0.194	0.435±0.149
M2-PRNet	MD75-1ns	0.695±0.249	0.804±0.297	0.769±0.048	0.694±0.123
GVP	MD75-10ns	1.048±0.142	1.310±0.232	0.346±0.286	0.250±0.245
IPA	MD75-10ns	1.079±0.295	1.337±0.419	0.393±0.328	0.365±0.288
CoPRA	MD75-10ns	0.961±0.277	1.177±0.357	0.551±0.203	0.420±0.155
M2-PRNet	MD75-10ns	0.702±0.269	0.812±0.305	0.752±0.056	0.694±0.130

Table S10. Experimental and M2-PRNet-predicted binding free energies and interface properties of VEGF165-RNA complexes. RNA interface coverage represents the fraction of RNA nucleotides containing at least one heavy atom within 5 Å of a protein heavy atom. Interface confidence was calculated as the mean pLDDT of protein and RNA residues located at the predicted interface.

Aptamer	Experimental ΔG (kcal/mol)	Predicted ΔG (kcal/mol)	RNA interface coverage (%)	Interface area (Å²)	Interface confidence
t44-OMe	-14.058	-10.183 ± 0.168	70.4 ± 19.1	1129 ± 337	53.6 ± 1.4
t22-OMe	-13.830	-10.211 ± 0.259	65.2 ± 10.2	1118 ± 396	52.8 ± 3.4
t2-OMe	-13.480	-10.290 ± 0.201	57.2 ± 5.2	1165 ± 248	54.0 ± 2.1
scr-t44	-8.803	-9.840 ± 0.103	46.7 ± 7.7	773 ± 145	52.3 ± 1.3

Table S11. Ablation study on PRA310 and PRA201.

Model Variant	*PRA310*				*PRA201*			
	RMSE (↓)	MAE (↓)	PCC (↑)	SPCC (↑)	RMSE (↓)	MAE (↓)	PCC (↑)	SPCC (↑)
M2-PRNet	1.368 ± 0.139	1.078 ± 0.121	0.601 ± 0.056	0.612± 0.069	1.301± 0.265	1.037± 0.227	0.598± 0.089	0.627± 0.095
-atom	1.405 ± 0.168	1.103 ± 0.118	0.570 ± 0.036	0.575± 0.051	1.383± 0.279	1.108± 0.254	0.557± 0.080	0.562± 0.115
-residue	1.415 ± 0.136	1.122 ± 0.137	0.555± 0.066	0.552± 0.074	1.497± 0.231	1.216± 0.195	0.457± 0.099	0.465± 0.124
-view	1.380 ± 0.145	1.087 ± 0.149	0.580± 0.061	0.572± 0.082	1.377± 0.264	1.105± 0.249	0.557± 0.116	0.561± 0.111
-con	1.382 ± 0.164	1.077 ± 0.167	0.581± 0.068	0.572± 0.105	1.357± 0.279	1.083± 0.254	0.549± 0.080	0.569± 0.115
-fusion	2.286 ± 0.936	1.659± 0.597	0.343± 0.219	0.402± 0.212	2.777± 0.723	1.921± 0.339	0.253± 0.186	0.286± 0.161
+tri-con	1.390 ± 0.159	1.097± 0.166	0.570± 0.074	0.589± 0.098	1.336± 0.301	1.049± 0.242	0.564± 0.076	0.591± 0.071

note: "-atom", "-residue", and "-view" denote removing the atom-level graph branch, residue-level graph branch, and tri-view image branch, respectively. "-con" denotes removing the contrastive learning objective. "-fusion" denotes replacing GateFusion with simple feature concatenation rather than removing feature fusion entirely. "+tri-con" denotes replacing the proposed pairwise contrastive learning objective with a tri-modal contrastive objective that jointly aligns atom-level graph, residue-level graph, and tri-view image representations. ↓ indicates lower is better, ↑ indicates higher is better.

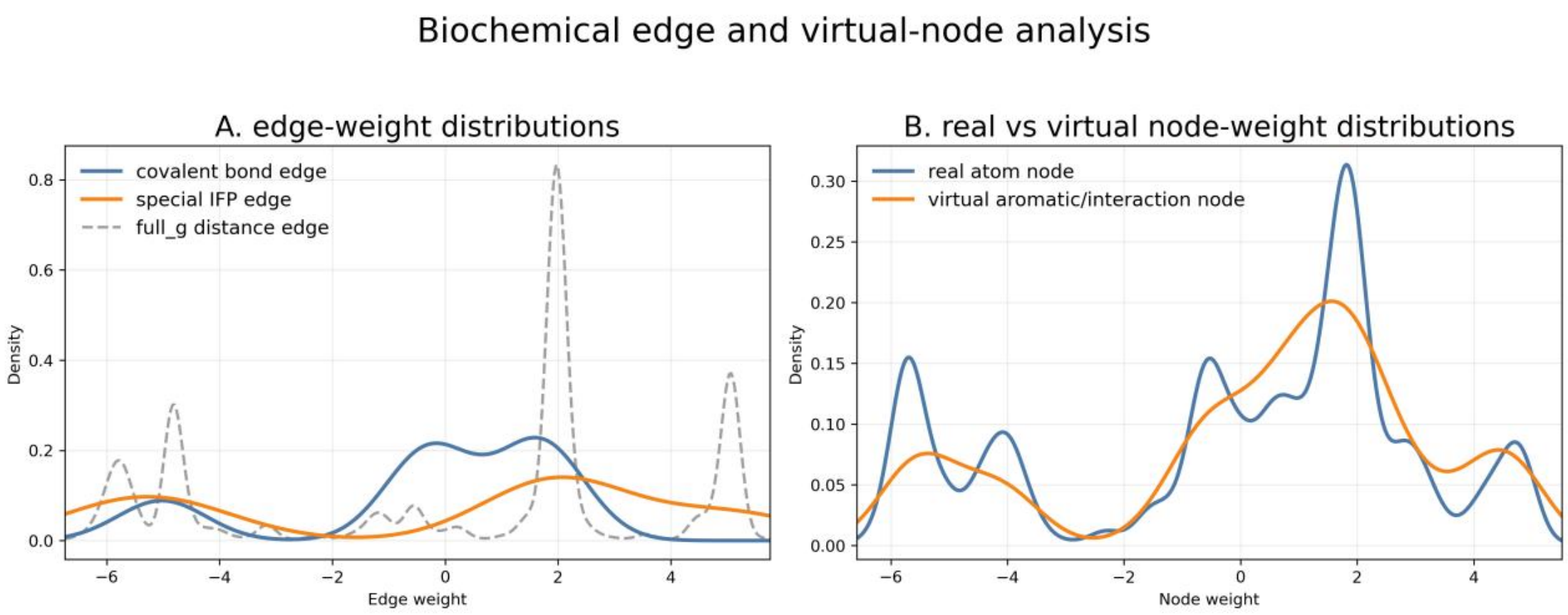

Fig. S1 Learned weight distributions of biochemical edge types and virtual nodes in the atom-level graph. (A) Distributions of learned edge weights for covalent bond edges, IFP-based interaction edges, and full-graph distance edges. Covalent bond edges and IFP-based interaction edges show relatively smooth and broadly similar distributions, whereas full-graph distance edges exhibit sharper local peaks, consistent with a more geometry-driven attention pattern. (B) Distributions of learned node weights for real atom nodes and virtual aromatic/interaction nodes. The comparable non-zero distributions indicate that virtual aromatic nodes contribute to message passing at a scale similar to real atom nodes.

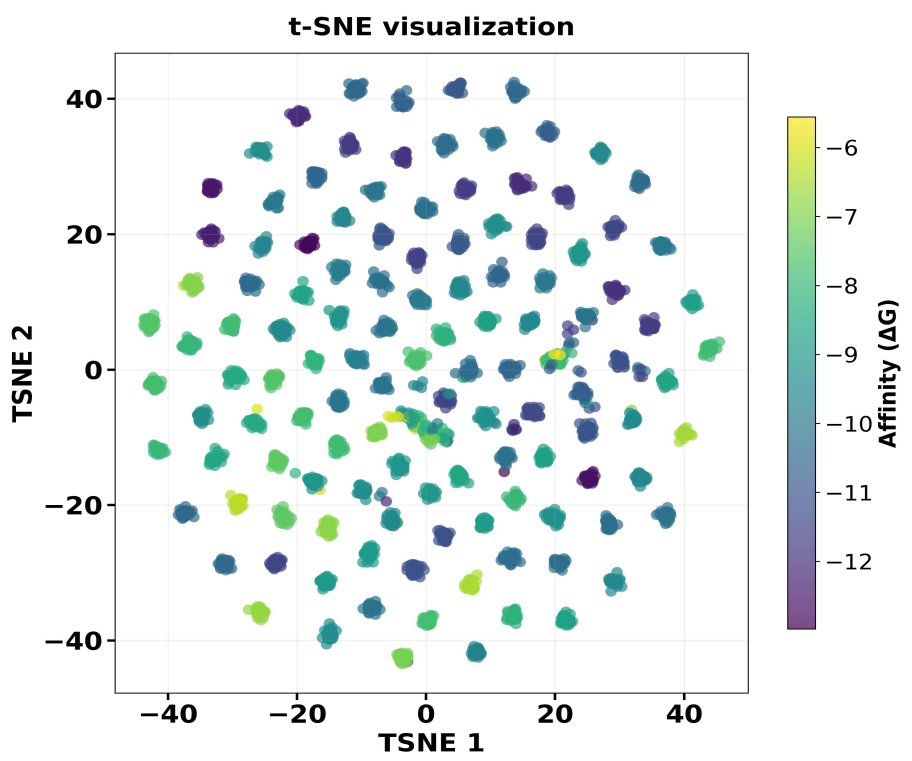

(a) Atomic feature

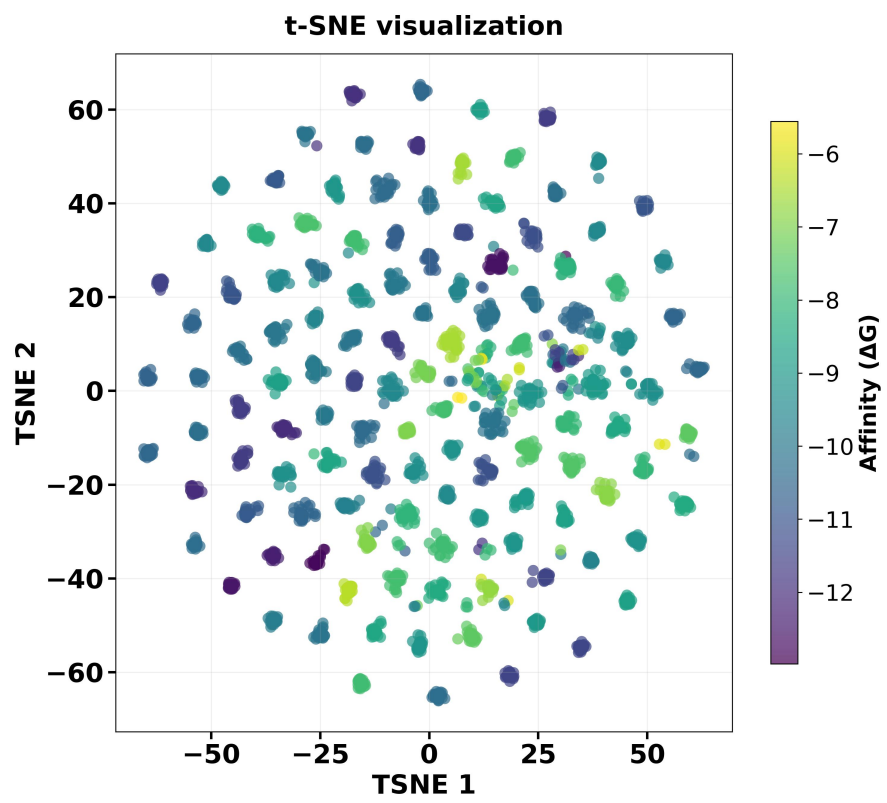

(b) Residue-level feature

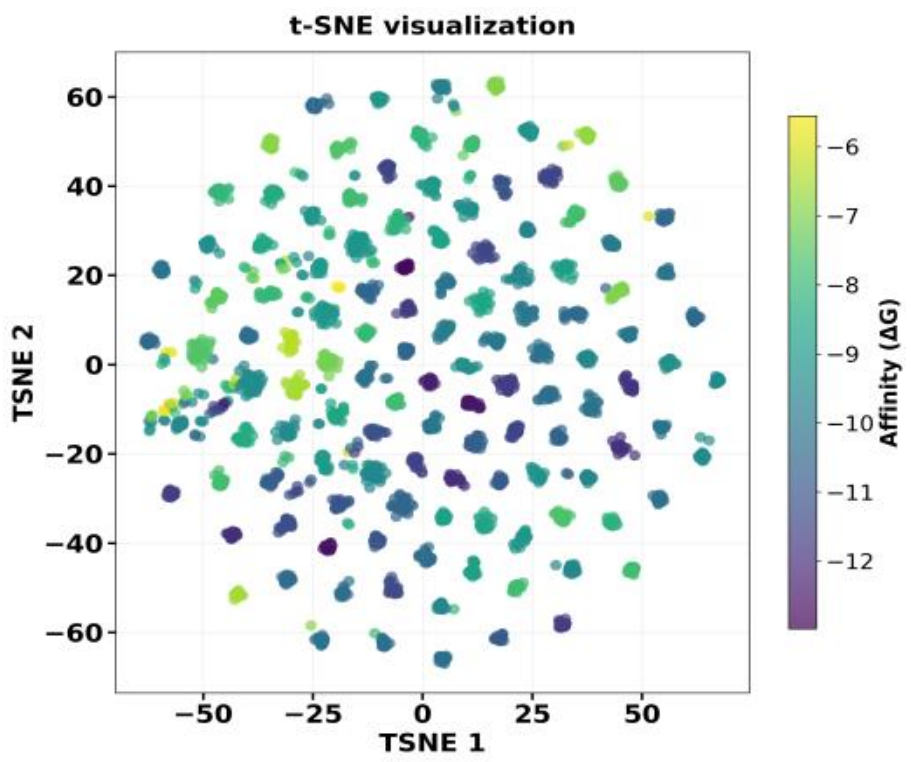

(c) Image feature

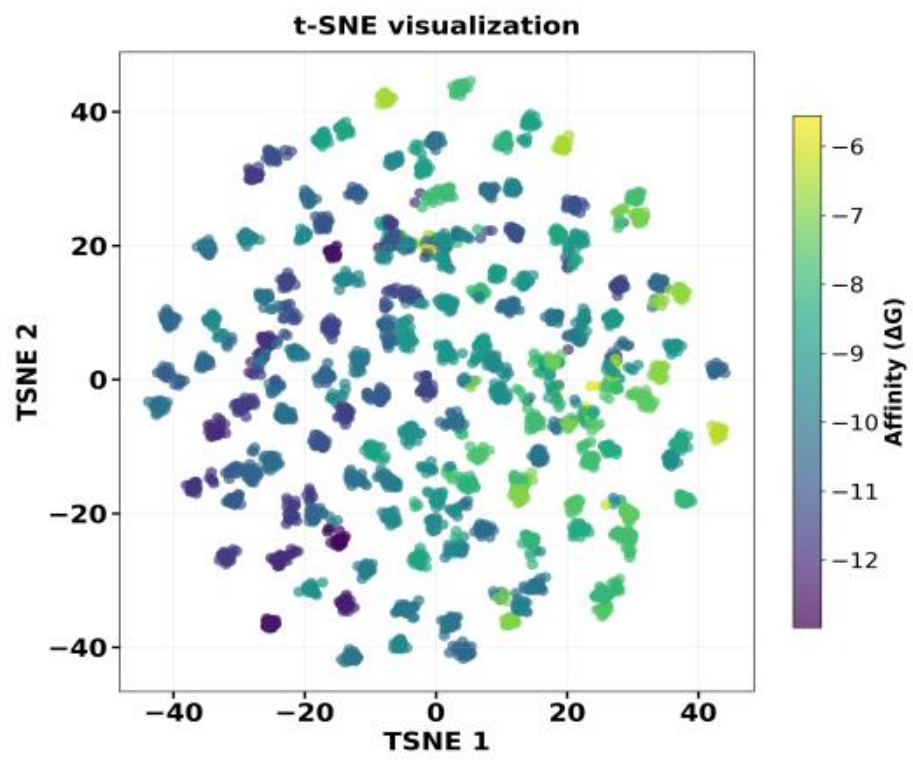

(d) Fused feature

Fig. S2 t-SNE visualization of feature distributions at different representation levels.

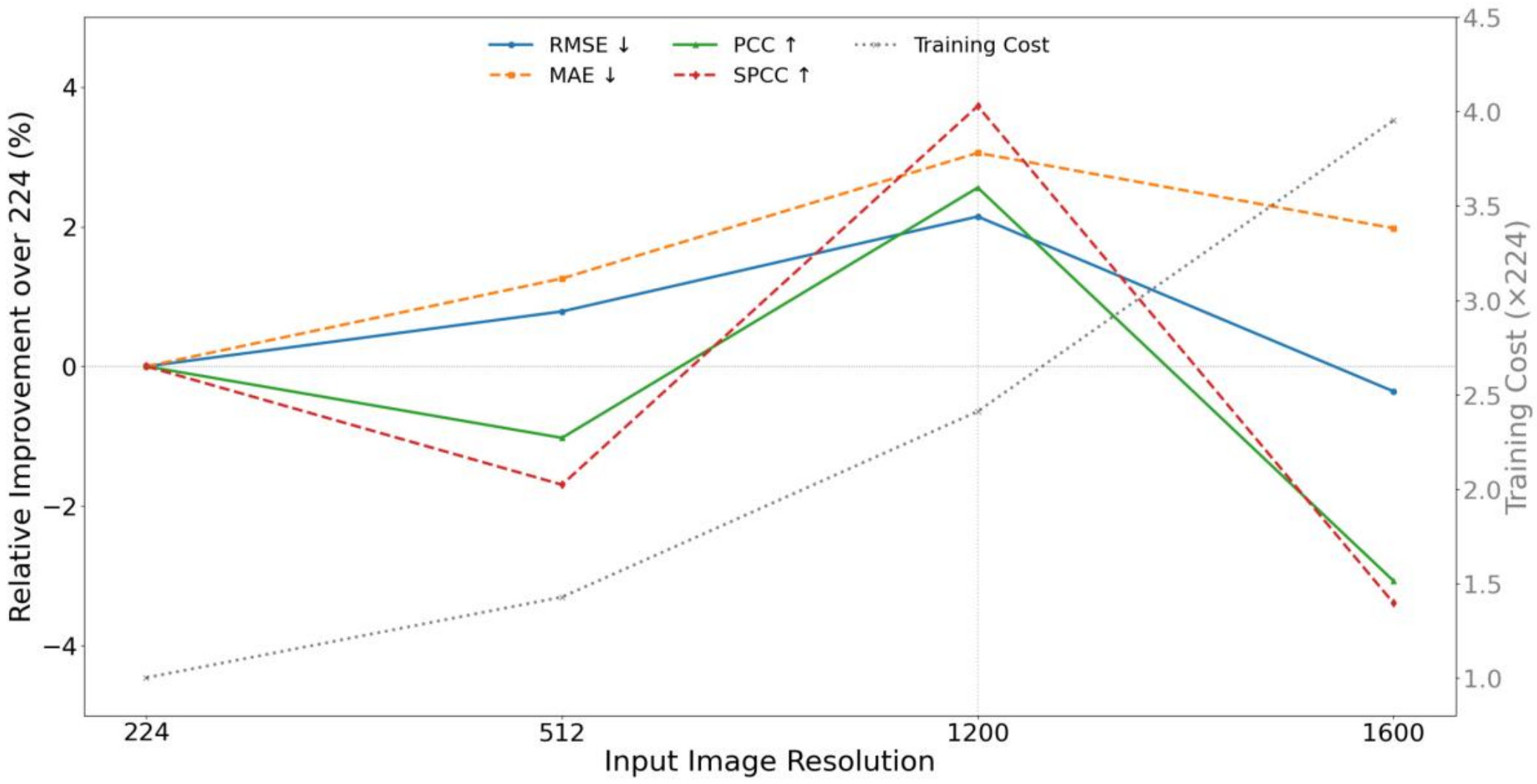

Fig. S3 Impact of tri-view image resolution on model performance and computational cost. Increasing the resolution improves prediction accuracy, with diminishing returns at higher resolutions, while computational cost increases steadily. Relative changes in RMSE, MAE, PCC, and SPCC are reported with respect to the 224 baseline, and normalized training cost is shown on the secondary axis.
